# Inferring Interaction Force from Visual Information without Using Physical Force Sensors

**DOI:** 10.3390/s17112455

**Published:** 2017-10-26

**Authors:** Wonjun Hwang, Soo-Chul Lim

**Affiliations:** 1 Department of Software and Computer Engineering, Ajou University, 206 Worldcup-ro, Yeongtong-gu, Suwon 16499, Korea; wjhwang@ajou.ac.kr; 2 Department of Mechanical, Robotics and Energy Engineering, Dongguk University, 30, Pildong-ro 1gil, Jung-gu, Seoul 04620, Korea

**Keywords:** deep learning, force estimation, interaction force, vision

## Abstract

In this paper, we present an interaction force estimation method that uses visual information rather than that of a force sensor. Specifically, we propose a novel deep learning-based method utilizing only sequential images for estimating the interaction force against a target object, where the shape of the object is changed by an external force. The force applied to the target can be estimated by means of the visual shape changes. However, the shape differences in the images are not very clear. To address this problem, we formulate a recurrent neural network-based deep model with fully-connected layers, which models complex temporal dynamics from the visual representations. Extensive evaluations show that the proposed learning models successfully estimate the interaction forces using only the corresponding sequential images, in particular in the case of three objects made of different materials, a sponge, a PET bottle, a human arm, and a tube. The forces predicted by the proposed method are very similar to those measured by force sensors.

## 1. Introduction

Human sensations during interaction with the physical world through tools or the skin are rich and varied. A person feels physical properties, such as strength, force, texture, and temperature, when holding objects in their hand or pressing them. This information is used when a person handles, grasps, and picks up various types of objects. For example, when picking up a variety of rigid objects, such as paper cups or glass cups, a person recognizes the physical properties of the object and handles the object according to that information. Another example is that surgeons feel the interaction force when they palpate organs during medical examinations, and pull thread using forceps during endoscopic surgery.

Recently, with the development of the robot industry, it has become important to use this information to help robots interact with the physical environment by sensing the properties of objects during their interaction. Service robots located in a human environment should be able to perform dexterous manipulation tasks in various conditions. Several studies have shown the interaction between a robot and its physical environment by sensing the physical properties, such as force [1,2,3], texture [4,5,6], and object shape [7], using various sensors. In particular, the main physical property that a robot grasping and interacting with objects needs to sense is the interaction force. For measuring this interaction force during the robot’s interaction with the environment, a tactile sensor [8,9] is used to sense a small force, such as a human skin sensation, and a force/torque sensor [10,11] to sense a larger force, such as a human kinesthetic force. Operations involving picking up an object by hand require a richer tactile and kinesthetic sense than that which the current systems provide in order to achieve human-level performance [12]. Furthermore, the physical sensors developed thus far have limitations in terms of their implementation, such as high cost or difficulty in attaching them to a real physical robot and other systems. In the case of a surgical robot, to measure the interaction force of forceps, various types of sensor, such as capacitive [13], piezoresistive [14], optoelectric [15,16], and strain gauge sensors [17], are used. However, a commercially available surgical robot is controlled remotely without haptic feedback, because it is difficult to attach a force measurement sensor to the forceps due to limited space, safe packaging, warm gas sterilization, and error-inducing EMI from electrocautery. Therefore, surgeons estimate the interaction force through a monitor when using this type of robot.

To reduce these constraints, many studies have been conducted on sensing interaction force without using a force/torque or tactile sensor. Geravand et al. [18] proposed a signal-based approach to whole body collision detection, robot reaction, and human-robot collaboration that is applied in the case of industrial manipulators with a closed control architecture by using the motor position, velocity, and motor currents, and without the use of additional sensors. Mattioli and Vendittelli [19] suggested a method for reconstructing interaction forces and localizing the contact point for humanoids under a static hypothesis based on the joint torque. Li and Hannaford [20] suggested a method for sensorless gripping force estimation of forceps with an encoder and joint torque sensing, based on the characteristics of elongated cable-driven surgical instruments.

Recently, an interaction force estimation technique without a force sensor was developed based on a depth camera or a camera and joint torque. Margrini et al. [21] developed a comprehensive approach using an RGB-D camera and joint torque for detecting, estimating, and handling dynamic force interactions that may occur at any point along the robot structure, to achieve an effective physical human-robot collaboration. As the use of deep learning technology is widespread in various research domains, it has been used to study the recognition of physical properties when a robot interacts with a physical object. Aviles et al. [22,23] showed a method of applied force estimation that uses a stereo camera in a surgical robotic system. A three-dimensional (3D) artificial heart surface is reconstructed from the projections of homologue points on the left and right lattices defined for each stereo-pair image, and supervised learning is applied to estimate the applied force and provide the surgeon with a suitable representation of it. In addition to robotics research, image-related research studies have also been conducted to predict interaction force through visual information. Zhu et al. [24] suggested a method of inferring interaction forces between a human body and a real-world object from video clips; however, they took into account physical quantities generated from 3D modeling. Pham et al. [25] showed a method for estimating contact forces during hand-object interactions that relies solely on visual input provided by images captured by a single RGB-D camera of a manipulated object with known geometrical and physical properties. Fermuller et al. [26] predicted actions in dexterous hand motions by using deep learning methods. Their method predicts different manipulation actions on the same object, such as “squeezing”, “flipping”, etc., by analyzing images. They also predicted the forces on the finger tips using the network. They predicted the force variations, but the absolute value of the force was not successfully estimated.

The objective of this study was to investigate the possibility of sensing the interaction force using a single camera, without using a physical tactile or force/torque sensor. A camera is a type of touchless sensor. Thus, no abrasion issues caused by long-term usage are involved, as compared to touchable sensors, e.g., tactile sensors. For this purpose, we propose that recurrent neural networks (RNNs) with fully-connected (FC) units are applicable to visual time-series modeling for force estimation and that learned temporal models can provide accurate force estimation by using sequential images, without requiring the use of physical force sensors. Specifically, the proposed end-to-end deep learning method allows the mapping of models from image pixels to an interaction force to be learned using long short-term memory (LSTM) [27]. The main contribution of this paper is that it presents the first investigation of the possibility that long-range learning deep models can infer interaction forces from the data of a vision sensor without requiring the use of physical force sensors. Unlike previous studies [21,22,23,24,25], in our study we used only 2D sequential images, not 3D models, for calculating the graphical models in order to measure the physical interactions between the objects. However, the proposed method involves deeper neural networks for learning straightforwardly the interaction forces between objects from 2D images. We performed comprehensive evaluations on three different materials (a sponge, PET bottle, and a living organism, an arm) and, in addition, we experimented with various condition changes of light and poses using a tube object, to demonstrate that the proposed method is capable of estimating the precise interaction force from the visual information.

This rest of this paper is organized as follows: In Section 2, we describe the RNNs on which the force estimation method is based. In Section 3, we describe the basic configuration of the proposed model architecture. In Section 4, we describe the database collection method and the experimental results, and present the discussion. We finally draw our conclusion and note the scope of further study in Section 5.

## 2. Recurrent Neural Network: Long Short-Term Memory

The RNN was basically designed to process time-series data [28,29,30] in applications such as speech and text recognition. For this purpose, it uses the internal memory to process arbitrary sequences. The convolutional neural network (CNN) [31], however, has been studied for processing fixed-size images and is a type of feed-forward neural network. Recently, the RNN has achieved good performance in various sequential data-based applications, but the problem of the vanishing and exploding gradient remains unresolved [32], which makes learning long-term dynamics difficult. To overcome this problem, LSTM [27] was proposed, which incorporates memory units and gate functions: a forget gate $f_{t}$ to control how much information from the past hidden state $h_{t-1}$ is preserved, an input gate $i_{t}$ to control how much the current input $x_{t}$ updates the memory cell $c_{t}$, an output gate $o_{t}$ to control how much information of the memory is fed to the output, and an input modulation gate $g_{t}$ to modulate the current input and the past hidden state before updating the memory cell. The memory cell $c_{t}$ preserves the state captured by the load cell of a sequence and updates the current hidden state unit with the output gate. The LSTM updates for the time step t given inputs, such as $x_{t}$, $h_{t-1}$, and $c_{t-1}$. The corresponding equations are:

(1) $$i_{t}=\sigma \left(W_{xi}x_{t}+W_{hi}h_{t-1}+b_{i}\right),$$

(2) $$f_{t}=\sigma \left(W_{xf}x_{t}+W_{hf}h_{t-1}+b_{f}\right),$$

(3) $$o_{t}=\sigma \left(W_{xo}x_{t}+W_{ho}h_{t-1}+b_{o}\right),$$

(4) $$g_{t}=tanh\left(W_{xc}x_{t}+W_{hc}h_{t-1}+b_{c}\right),$$

(5) $$c_{t}=f_{t}⊙c_{t-1}+i_{t}⊙g_{t},$$

(6) $$h_{t}=o_{t}⊙tanh\left(c_{t}\right),$$

where $\sigma \left(x\right)={\left(1+e^{-x}\right)}^{-1}$ is the sigmoid activation, $tanh\left(x\right)=\frac{e^{x}-e^{-x}}{e^{x}+e^{-x}}=2\sigma \left(2x\right)-1$, and ⊙ denotes element-wise multiplication. All the weights, W, and biases, b, of the network are learnt jointly on training data. In this study, we aimed to investigate the possibility of predicting the interaction force using only sequential images. We propose an LSTM-based deep learning architecture for this challenge.

## 3. Proposed Deep Learning Model Architecture

**Figure 1:**
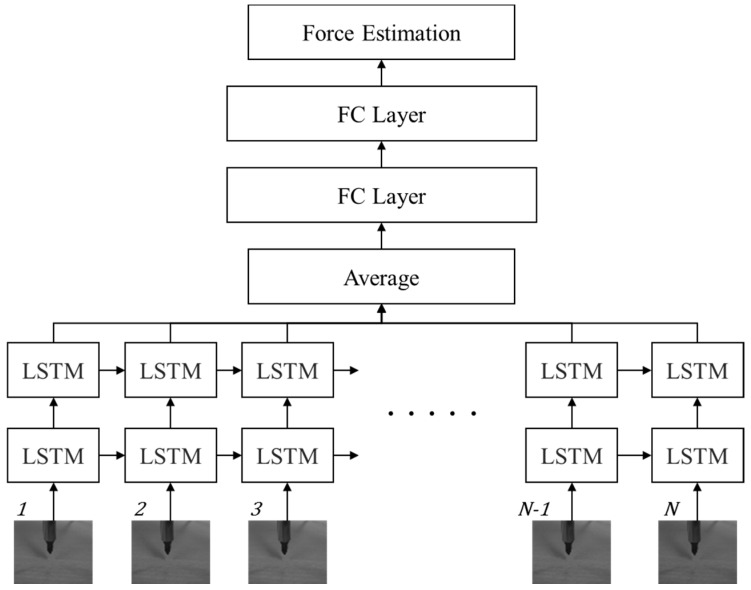
Architecture of the proposed long short-term memory (LSTM) model for estimating interaction force using only sequential images. Two layer-based LSTMs are used for accurate sequential learning and the N sequential outputs of the LSTMs are processed to estimate the interaction force through two stacked fully connected layers.

In this section, we propose a novel deep learning framework for estimating the interaction forces using only sequential images. It should be noted that we do not use a physical force sensor and additional depth sensors to collect additional measurements. The main learning task is the prediction of a single static value, the interaction force, at a specific moment, from the sequential visual inputs. The motivation to develop the proposed method is as follows. Humans, when in contact with an object, can infer how the forces applied to it interact with each other by observing the change in the shape of the object. This is largely because they have already gained sufficient experience of interaction forces during their life. From this viewpoint, we propose a vision-based interaction force estimator that uses the deep learning method. The overall view of the proposed LSTM-based framework is shown in Figure 1. It contains two parts: the LSTM part models the relationship between the image and the interaction force using the total N sequential images and the FC layer part learns the processing model from the LSTM output to obtain an accurate estimation of the interaction force in a straightforward manner. The temporal aspect of the proposed method is important. In general, the situation of loading and unloading have an effect on the relationship between contact forces and indentation. This hysteresis damping generates different contact forces at the same deformation [33]. Due to the hysteresis, the force is measured differently at the same deformation according to the loading and unloading conditions.

To avoid the obstacles of background clutter simply, we crop only the center region from an input image and normalize it to a fixed resolution image W×H, where W is the width and H is the height, with a single gray channel. We directly convert the gray image into a visual input vector $x\in {ℜ}^{W\times H}$ and the total N sequential normalized images are passed into an LSTM sequence learning module. The proposed model stacks one more LSTM on another to gain more accurate results, because the recent deep model for object recognition [34] suggests that deeper layers usually lead to more accurate classification results. We apply a late-fusion method to merge the N step LSTM output for generating the input of the FC layers. It should be noted that the proposed method predicts the final static output, e.g., the interaction force value, with the sequential inputs. In this respect, to achieve better results, we add up two FC layers with N LSTM outputs. The basic assumption of the proposed method is that, if sufficient preceding input data exists, the most accurate result can be predicted using FC layers in the final time step. In RNNs, arbitrary input and output sizes are commonly handled, but, in this method, we take a fixed-size input for predicting the interaction force at the end of the network operation. Our main task is not to process arbitrary sizes of inputs and outputs, but to predict a single value using the sequential inputs. In this case, we can easily train the network with fixed-size inputs, because we can remove the uncertainty regarding the sizes of inputs and outputs in the training stage. We handle the sequential inputs with LSTM modules. For the first N time steps, the LSTM processes the total N inputs. Since FC layers need fixed-length input from LSTMs, until N time steps are executed, there are no prediction results.

The predicted interaction force, $y=f_{lstm}\left(x\right)$, $x=\left\{x_{1},x_{2},\ldots ,x_{N}\right\}$, is computed by taking the mean square error (MSE). The difference between the target and the estimated value is minimized based on MSE criteria. It is represented as:

(7) $$L\left(\tilde{y},y\right)=\frac{1}{N}\overset{N}{\underset{i=1}{\sum }}{\left({\tilde{y}}_{i}-y_{i}\right)}^{2}$$

where $\tilde{y}$ and y are the predicted interaction force and the ground-truth, respectively.

## 4. Experimental Results and Discussion

In this section, we first describe the new database collected for the interaction force estimation and the experimental settings for evaluating the proposed network architecture, and then we compare the estimated results of the proposed method and the ground truth (GT) captured by the load cell (model BCL-1L, CAS) for three different material-based objects: a sponge, PET bottle, and human arm. We also present additional experiments conducted using a tube under the different conditions such as illumination changes and posed load cell movements, to better analyze the proposed method.

### 4.1. Datasets and Implementation Details

**Figure 2:**
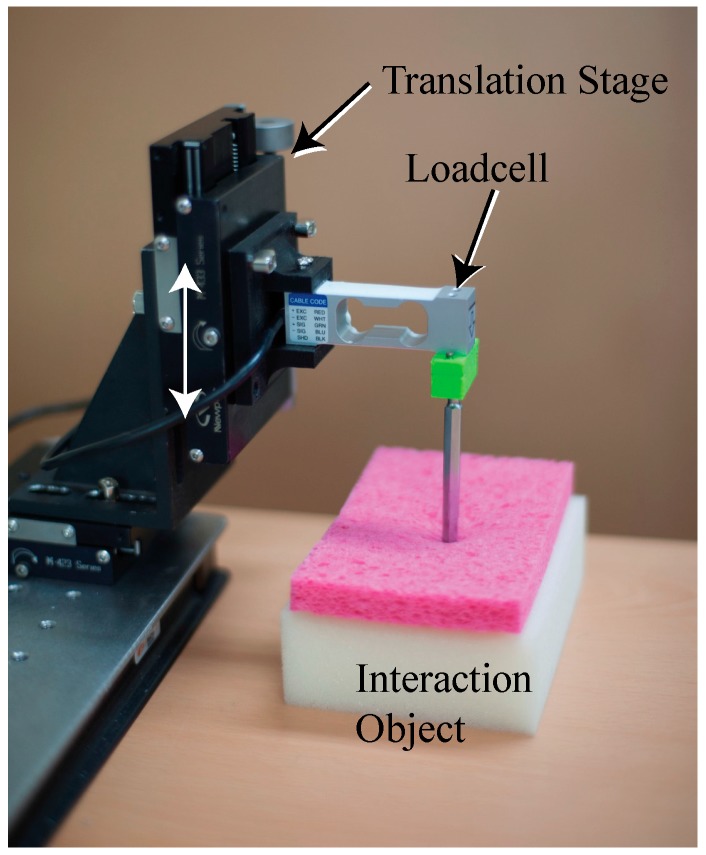
Interaction force measurement system with the load cell and translation stage.

*Interaction force measurement instrument:* To facilitate the proposed method, we constructed new datasets by capturing sequential images using a 149 Hz camera (Cameleon3, CM3-U3-13Y3C-CS, Pointgrey). At the same time as taking an image, we measured and recorded the interaction force in each image using a load cell. As shown in Figure 2, in the experimental interaction force measurement system a rod that interacts with the load cell allows the device to touch the interaction object. The load cell is fixed to the translation stage, the movement of which is limited to the *z*-direction, to measure the interaction force. The database was created by moving the translation stage to the arbitrary length of motion in the experimental device shown in Figure 2 and recording the image and force at that time.

**Figure 3:**
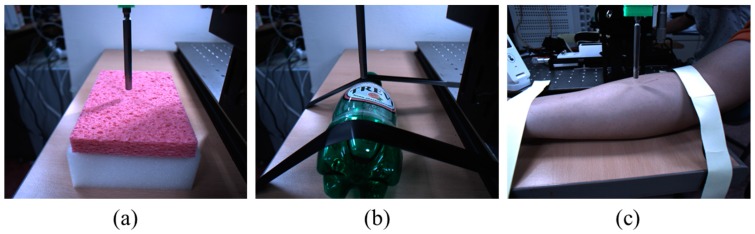
Experimental conditions with the load cell at the *z* axis movable translation. Test objects are (**a**) a sponge; (**b**) a PET bottle; and (**c**) a human arm.

*Training, validation, and test protocols:* To build the training and test protocols, we collected approximately 6000 sequential images using an RGB camera and the corresponding interaction forces captured by the load cell in the *z* direction. Example images, comprising 1280 × 1024 resolution-based images with RGB color, are shown in Figure 3. We collected the images of objects constructed from three different materials, a sponge, PET bottle, and human arm, for the training and test sets. One image set consists of four contacts with the material; a total of 14 sets were collected for each material. To evaluate the proposed method, we basically used the repeated random sub-sampling validation protocol [35] with 14 image sets. An advantage of this method is that the proportion of training and test split is not dependent on the number of the image sets. We wanted to secure a sufficient number of test images (e.g., four sets with approximately 1900 images) for confirming the accuracy of the proposed method. First, we randomly selected four sets as the test set from the 14 sets, and the other 10 sets were used as the training set. We also split the training images into training and validation sets for avoiding overfitting. Further, we randomly selected two sets from the 10 training sets as the validation set. We used the validation set that the training algorithm does not observe and the test set is not used in any way to make choices about the model and its hyperparameters. The validation set is only used for guiding the selection of the hyperparameters [36]. After hyperparameter optimization is complete, the generalization error could be estimated using the test set. Using this procedure, we have five different training, validation, and test sets. The final test error is estimated by taking the average test error across five trials using five different selected training, validation, and test sets. Table 1 describes the image numbers for the three different material-based objects.

**Figure 4:**
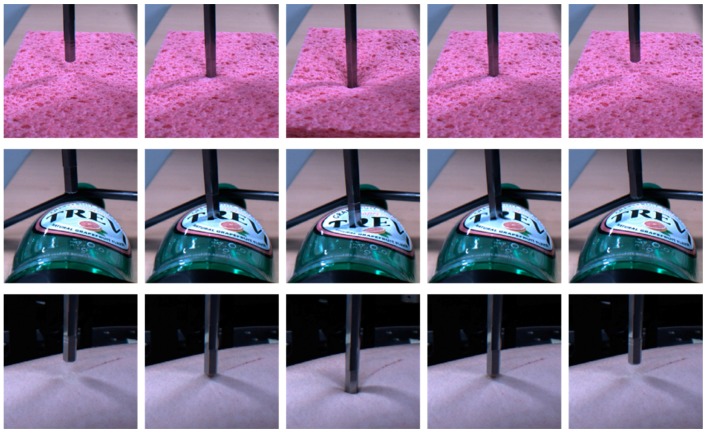
One cycle of the cropped contact images. The first row shows images of the sponge, the second row images of the PET bottle, and the final row images of the human arm.

*Network implementation:* Our implementation for estimating the interaction force is illustrated in Figure 1. The network structure comprises two stacked LSTM layers and two dense FC layers. From this network, we finally estimate the interaction force using mean square error (MSE) as the network loss. The sequential input images are cropped and normalized from 1280 × 1024 pixel-based images to 20 × 20 pixel-based gray images. Examples of the collected images are shown in Figure 4. Histogram equalization is used as the preprocessing procedure for compensating the illumination changes. The cropped input image is changed to a 400-dimensional input vector for the network. For building the precise estimator, we use the 40 time step for the LSTM inputs. The number of the weight parameters for the two stacked LSTMs are 10 and 5, respectively, and the two FC layers consist of 10 and 10 weight parameters, respectively. Between the FC layers, there is a dropout unit, the value of which is 90%. The basic architecture of the proposed method is summarized in Table 2. We initialize the weights randomly and train all network weights from scratch. We use an Adagrad optimizer for fast calculation, and the models are trained with a mini-batch size of 100 on four TitanX GPUs. The learning rate is 0.1 and the final models are trained for up to 2500 epochs.

### 4.2. Investigation of Different Material-Based Objects

In this section, we describe three different experiments conducted for validating that the proposed method is effective. We trained the proposed RNN models independently using the target training set and conducted a test with the corresponding test set.

#### 4.2.1. Material 1: Sponge

We first evaluated the proposed network using the sponge dataset, because, as shown in Figure 4, the shape changes of the sponge according to the force are more apparent than those of the other materials. It should be noted that, since a sponge is highly elastic, it has the characteristic that a relatively broad neighboring area is pushed down together with the point at which the force is applied. As can be seen in the performance comparison in Table 3, the average MSE and peak signal-to-noise (PSNR) range from 0.0021 to 0.0039 and from 24.32 dB to 26.86 dB, respectively. The average difference between the estimated force and the ground-truth is small. A healthy person can easily sense the changes in the force with the index finger when the interaction force changes by 10% as compared to the previous one at a force range of 0.5–200 N [37,38]. For example, the start force against the index finger is 10 N and the difference is greater than 1 N at the subsequent time; the person can feel the force change intuitively. This threshold is increased to 15–27% under a 0.5 N force perception. Therefore, normally persons cannot recognize the difference between the interaction force estimated by the proposed method and the real value captured by the load cell.

**Figure 5:**
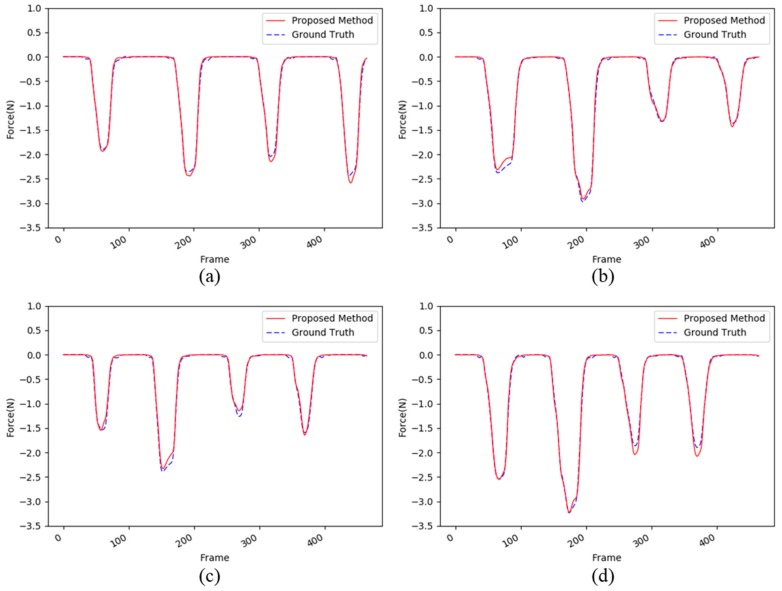
An example of the four evaluations from (**a**–**d**) for sponge dataset. The red dotted lines and the blue lines show the force estimated by the proposed method and the ground truth captured by the load cell, respectively.

Figure 5 shows the detailed performance comparisons from the first test set to the final one. In each test set, the maximum interaction forces have values varying from −1 N to −3.5 N. However, the proposed method can successfully estimate the interaction forces using only images. In summary, we conclude that the proposed deep learning network can estimate the interaction force using as input the apparent change in the sequential images.

#### 4.2.2. Material 2: PET Bottle

**Figure 6:**
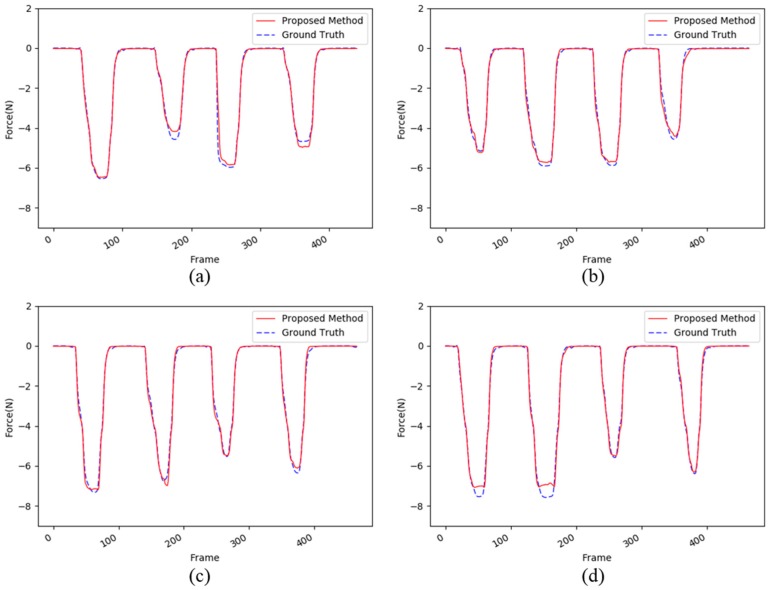
An example of the four evaluations from (**a**–**d**) for the PET bottle dataset. The red dotted lines and the blue lines show the force estimated by the proposed method and the ground truth captured by the load cell, respectively.

Next, we evaluated the proposed method using the PET bottle test set. Unlike the sponge, the PET bottle is not flat, but it has a unique shape. As shown in Figure 4, the image changes of the PET bottle due to the interaction force are quite different from those of the sponge. This is largely because the PET bottle has various curvatures as compared with the sponge, which resembles a square box. The performance difference between the materials, sponge and PET bottle, can be seen clearly in Table 4 as compared with Table 3. The average MSE and PSNR range from 0.018 to 0.042 and from 13.83 dB to 17.56 dB, respectively, and these results are not good as compared with the results of the sponge test sets. However, as shown in Figure 6, the proposed method accurately estimates the interaction force using only images, except for the peak of the interaction force. It should be noted that the first two maximum peaks in Figure 6d are not relatively accurately estimated. This is largely because the image changes of the pressed object, i.e., the PET bottle, are not clearly visible as a result of self-occlusion. However, in the other cases, the estimated forces are very similar to the ground-truth.

#### 4.2.3. Material 3: Human Arm

**Figure 7:**
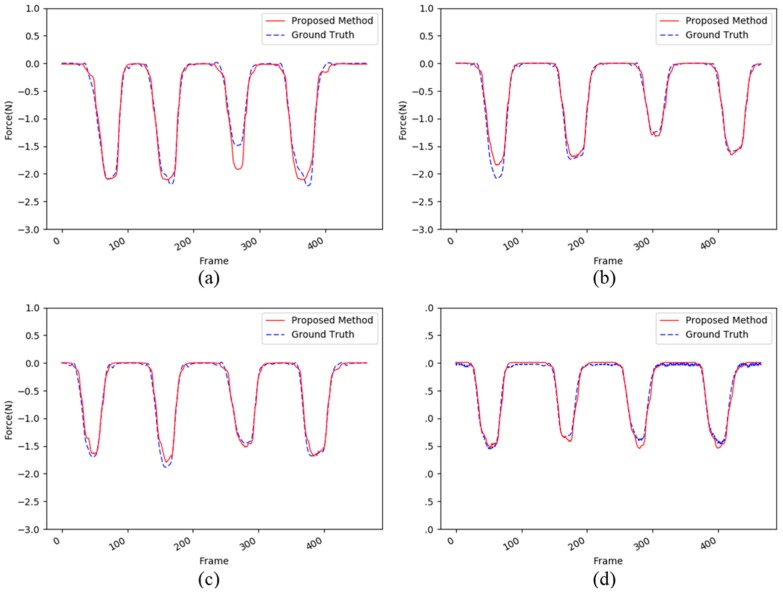
An example of the four evaluations from (**a**–**d**) for the human arm dataset. The red dotted lines and the blue lines show the force estimated by the proposed method and the ground truth captured by the load cell, respectively.

This experiment was conducted to validate the proposed method using the human arm test set in order to evaluate complex situations. As shown in Table 5, our proposed method shows good performances in general for the human arm test set. For example, the average MSE and PSNR values range from 0.007 to 0.014 and from 18.74 dB to 22.17 dB, respectively. Figure 7c shows the best performances; all the forces are relatively well estimated by the proposed method. However, Figure 7a,b,d show atypical patterns for the human arm as compared with the other materials. This is largely because the human arm is a living tissue and the resilience of the arm can be changed by the muscle. When we captured images for the database, the subject tried not to move the muscles, but it is not possible to keep the arm motionless, unlike general objects such as a sponge and PET bottle. Therefore, there are some differences in the third touch in Figure 7a and the first touch in Figure 7b.

#### 4.2.4. Discussion

**Figure 8:**
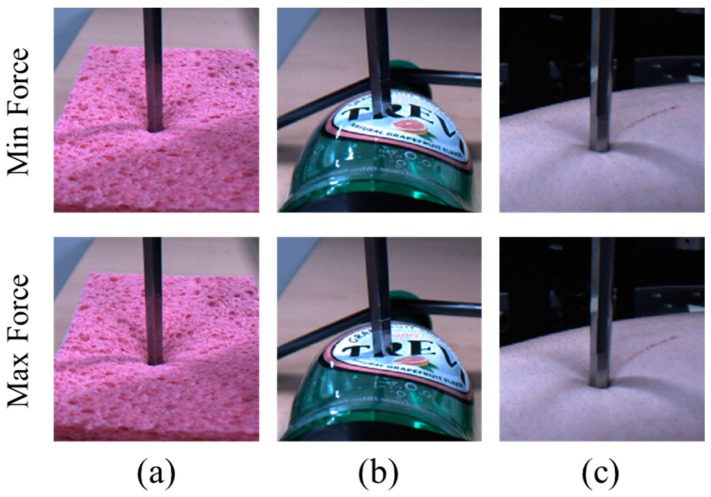
Example images when the minimum or maximum interaction force is applied. From left to right, (**a**) sponge; (**b**) PET bottle; and (**c**) human arm images are shown.

Now we compare the characteristics of the three different materials in detail. First, Table 6 shows the average MSE and PSNR across the three test materials. The best prediction accuracy is achieved by the sponge material, because the appearance of the object is changed by the external force more than that of the other materials and this is the key point for image-based interaction force estimation. However, the experiments using the PET bottle show the lowest accuracy, because some self-occlusion issues occurred. As shown in Figure 8b, the difference between the images when the maximum force or the minimum force was applied is not significant. Figure 8a,b shows the image differences according to the minimum and maximum forces. However, it should be noted that the average MSE for the PET bottle is only 0.031 and this accuracy in the image-based interaction force estimation is not low as compared with that of human force perception [37,38]. From these results, we can conclude that the proposed method yields a good accuracy for predicting the interaction force using only images.

**Figure 9:**
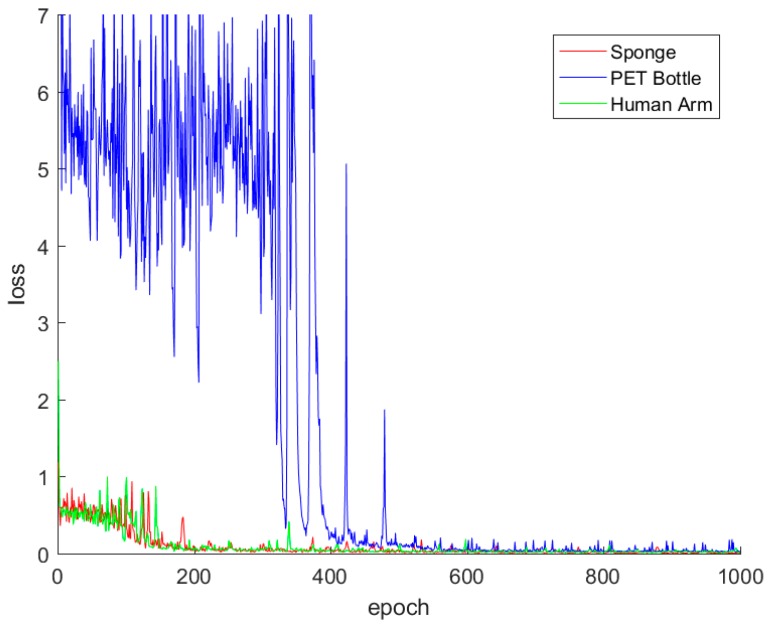
Training losses for the three different materials.

Figure 9 shows the differences in training loss in the training stages for the three different materials. It should be noted that the loss in the PET bottle training does not easily drop off when learning the network models as compared to that of the other objects. As from the results in Table 5, we can infer from these results that training the model for the PET bottle is not easy; however, after 500 epochs, the variance in the loss decreases rapidly.

### 4.3. Investigation of Different Variations

**Figure 10:**
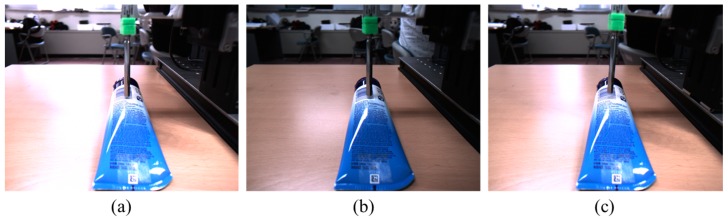
Example images of a tube are shown in (**a**) normal condition; (**b**) change in illumination; and (**c**) change in pose of the load cell movement (e.g., five degrees).

**Figure 11:**
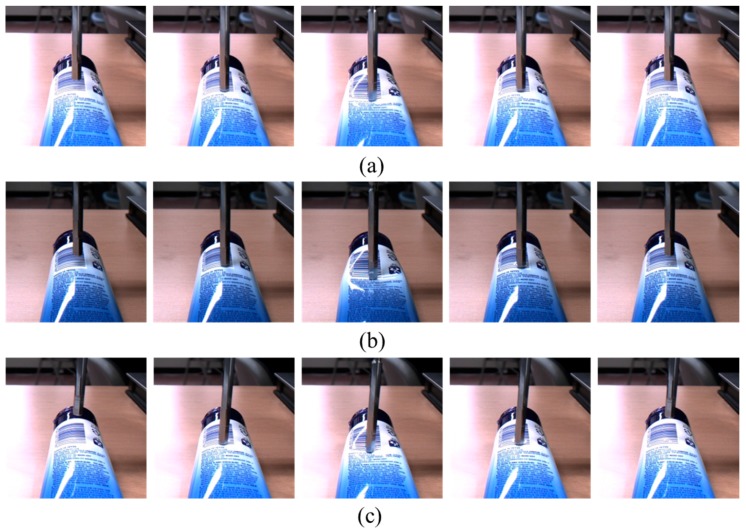
Example cropped images of a tube are shown (**a**) normal condition, in the first row; (**b**) light change, in the second row; and (**c**) pose change of the load cell movement, in the last row.

In this section, we present an evaluation of the performance changes of the proposed method according to the various external variations. For this experiment, we collected additional images using a tube. Unlike the experiments previously described, the experiment protocol here involves taking sequential images with a tube under the various environment changes, such as varying illumination and the posed load cell (or a different poking angle). The number of images is described in Table 7, and example images are shown in Figure 10. The cropped images under the different illumination conditions are also shown in the second row of Figure 11. The last row of Figure 11 shows the five-degree posed load cell used to apply a force on the material. In this evaluation protocol, we train a deep learning model using a normal set, a light change set, and a pose change training set (32 set = 8 set × 3 variation) simultaneously. We evaluate the performances of the proposed method by using the test sets.

Table 8 shows the accuracy of the proposed method according to the variations. In the case of the normal condition, the average MSE is 0.015 and it is relatively similar to the result of the human arm. However, the test results of the light and pose changes show average MSEs of 0.037 and 0.019, respectively. This result shows that the proposed method is robust to 5 degree pose variation but it is relatively weak for the light changes. One reason for this result is that the ratio of the training images, which is normal illumination, is larger than the light change training image, because the light condition of the pose change set is identical to that of the normal condition. Moreover, when the ceiling fluorescent lamp is turned off (e.g., light change set), the texture on the tube is not clearly visible. It leads to relatively degraded performance, because the proposed method depends on the visual information and produces the estimated results. However, note that the 0.037 average MSE is not a poor result compared with the result of the PET bottle. Overall, we validate the performances of the proposed method under the various conditions and conclude that the proposed method performs reasonably well for the different variations, such as light and pose changes.

## 5. Conclusions

In this paper, we proposed an interaction force estimation method that uses a vision sensor and does not require a force sensor. We proposed a deep learning method based only on sequential images for estimating the interaction force. We measured the force and recorded the images of three objects at the time when the touch occurred, by using the device described in this paper and a load cell, which moved only in the direction of pressing, where deformation occurs. We formulated an RNN-based deep model with fully connected layers for applying the database that we created. Extensive evaluations showed that the proposed learning models successfully estimate the interaction forces using only the corresponding sequential images for four different deformable object materials, a sponge, a PET bottle, an arm, and a tube. Our future plans include the development of a three-axis force estimation model that does not require an additional force sensor, but rather uses a vision sensor attached to the robot’s hand.

## Figures and Tables

**Table 1:** The collected sequential images in which each set consists of four touches with approximately 470 sequential images.

	Sponge	PET Bottle	Human Arm
Total number of Image	6651 (14 sets)	6568 (14 sets)	6624 (14 sets)

**Table 2:** Network architecture of the proposed method.

Force Estimation Model	
Layer Name	Layer Description
Input	Input 20 × 20 gray image
LSTM layer 1	Time step = 40, Input = 4000, Output = 10
LSTM layer 2	Time step = 40, Input = 10, Output = 5
Element-wise Mean	-
FC layer 1	10-fully connected, ELU (*α* = 1.0)
Dropout	Keep prop = 0.9
FC layer 2	10-fully connected, ELU (*α* = 1.0)
Linear Regression	Mean Squared Error

**Table 3:** Comparison of performances of the proposed method and the ground truth in the sponge test dataset.

Test Set	Average MSE	Average PSNR (dB)
1	0.0021	26.86
2	0.0039	24.32
3	0.0030	25.76
4	0.0035	24.52

**Table 4:** Comparison of the performances of the proposed method and the ground truth in the PET bottle test dataset.

Test Set	Average MSE	Average PSNR (dB)
1	0.040	14.02
2	0.018	17.56
3	0.025	16.09
4	0.042	13.83

**Table 5:** Comparison of performances of the proposed method and the ground truth in the human arm test dataset.

Test Set	Average MSE	Average PSNR (dB)
1	0.014	18.74
2	0.009	20.96
3	0.007	22.17
4	0.012	20.50

**Table 6:** Comparison of average performances for the three materials.

Material	Average MSE	Average PSNR (dB)
Sponge	0.0032	25.37
PET bottle	0.031	15.37
Human arm	0.010	20.59

**Table 7:** The number of images collected from a tube material under the different conditions.

	Normal	Light Change	Pose Change
Total number of Image	6086 (14 sets)	6085 (14 sets)	6096 (14 sets)

**Table 8:** Comparison of performances of the proposed method and the ground truth under the various conditions such as light and pose changes.

Test Set	Normal		Light Change		Pose Change	
	Average MSE	Average PSNR (dB)	Average MSE	Average PSNR (dB)	Average MSE	Average PSNR (dB)
1	0.017	18.06	0.030	15.46	0.027	15.99
2	0.019	17.85	0.037	14.48	0.015	18.25
3	0.017	17.81	0.037	14.80	0.017	17.88
4	0.010	20.06	0.044	13.73	0.017	18.36
Average	0.015	18.44	0.037	14.62	0.019	17.62
